# Distinct Occurrence of Proarrhythmic Afterdepolarizations in Atrial Versus Ventricular Cardiomyocytes: Implications for Translational Research on Atrial Arrhythmia

**DOI:** 10.3389/fphar.2018.00933

**Published:** 2018-08-21

**Authors:** Nils Bögeholz, Paul Pauls, Dirk G. Dechering, Gerrit Frommeyer, Joshua I. Goldhaber, Christian Pott, Lars Eckardt, Frank U. Müller, Jan S. Schulte

**Affiliations:** ^1^Clinic for Cardiology II – Electrophysiology, University Hospital Münster, Münster, Germany; ^2^Institute of Pharmacology and Toxicology, University of Münster, Münster, Germany; ^3^Heart Institute, Cedars-Sinai Medical Center, Los Angeles, CA, United States; ^4^Department of Cardiology, Schuechtermann-Klinik, Bad Rothenfelde, Germany

**Keywords:** afterdepolarizations, translational research, atrial cardiomyocytes, atrioventricular differences, atrial arrhythmia

## Abstract

**Background:** Principal mechanisms of arrhythmia have been derived from ventricular but not atrial cardiomyocytes of animal models despite higher prevalence of atrial arrhythmia (e.g., atrial fibrillation). Due to significant ultrastructural and functional differences, a simple transfer of ventricular proneness toward arrhythmia to atrial arrhythmia is critical. The use of murine models in arrhythmia research is widespread, despite known translational limitations. We here directly compare atrial and ventricular mechanisms of arrhythmia to identify critical differences that should be considered in murine models for development of antiarrhythmic strategies for atrial arrhythmia.

**Methods and Results:** Isolated murine atrial and ventricular myocytes were analyzed by wide field microscopy and subjected to a proarrhythmic protocol during patch-clamp experiments. As expected, the spindle shaped atrial myocytes showed decreased cell area and membrane capacitance compared to the rectangular shaped ventricular myocytes. Though delayed afterdepolarizations (DADs) could be evoked in a similar fraction of both cell types (80% of cells each), these led significantly more often to the occurrence of spontaneous action potentials (sAPs) in ventricular myocytes. Interestingly, numerous early afterdepolarizations (EADs) were observed in the majority of ventricular myocytes, but there was no EAD in any atrial myocyte (EADs per cell; atrial myocytes: 0 ± 0; *n* = 25/12 animals; ventricular myocytes: 1.5 [0–43]; *n* = 20/12 animals; *p* < 0.05). At the same time, the action potential duration to 90% decay (APD_90_) was unaltered and the APD_50_ even increased in atrial versus ventricular myocytes. However, the depolarizing L-type Ca^2+^ current (I_Ca_) and Na^+^/Ca^2+^-exchanger inward current (I_NCX_) were significantly smaller in atrial versus ventricular myocytes.

**Conclusion:** In mice, atrial myocytes exhibit a substantially distinct occurrence of proarrhythmic afterdepolarizations compared to ventricular myocytes, since they are in a similar manner susceptible to DADs but interestingly seem to be protected against EADs and show less sAPs. Key factors in the generation of EADs like I_Ca_ and I_NCX_ were significantly reduced in atrial versus ventricular myocytes, which may offer a mechanistic explanation for the observed protection against EADs. These findings may be of relevance for current studies on atrial level in murine models to develop targeted strategies for the treatment of atrial arrhythmia.

## Introduction

Triggered arrhythmia originates from early (EADs) and delayed (DADs) afterdepolarizations on the cellular level (Damiano and Rosen, 1984; Levine et al., 1985; January and Riddle, 1989). Underlying molecular mechanisms and principle concepts of proarrhythmic mechanisms have been well defined in ventricular cardiomyocytes in various experimental models (Pogwizd et al., 1999, 2001; Sipido et al., 2000; Pott et al., 2012). In the past two decades, murine models have been increasingly investigated in arrhythmia research due to their accessibility for genetic manipulation despite significant differences between mouse and human physiology (for review, see Nerbonne, 2014).

Yet atrial – or in clinical terms *supraventricular* – arrhythmias are much more prevalent than ventricular arrhythmias and attract great attention in clinical and translational research, especially in the case of atrial fibrillation (Kirchhof et al., 2016). However, the electrophysiological properties and underlying molecular mechanisms in atrial cardiomyocytes are considerably less defined as compared to ventricular cardiomyocytes, especially in smaller rodents like mice and rats (Walden et al., 2009). Nevertheless, murine models are frequently used to investigate proarrhythmic substrates or to evaluate potential antiarrhythmic strategies to counter atrial arrhythmia even on multicellular or whole-heart level (Bao et al., 2016; Syeda et al., 2016; Wan et al., 2016; Chang et al., 2017; Wang et al., 2017). Yet, there is a lack of studies, which directly compare proarrhythmic substrates and their molecular basis in atrial versus ventricular cardiomyocytes of murine models, which appears unexpected in the face of the high clinical and translational relevance of atrial arrhythmia and the widespread use of murine models in the research of atrial arrhythmia.

One might suppose that ventricular proneness toward proarrhythmic afterdepolarizations can simply be applied on the atrial level. However, this requires a consistent functional and structural analogy between atrial and ventricular cardiomyocytes, which is not the case. Previous work demonstrated substantial differences regarding the morphology and microarchitecture of atrial and ventricular cardiomyocytes (Bossen et al., 1981; Blatter et al., 2003; Brette et al., 2005; Walden et al., 2009; Bootman et al., 2011; Frisk et al., 2014). Thus, atrial myocytes rather resemble smooth-muscle myocytes due to the diminished sarcomere structure and a fusiform shape. Also, t-tubules as a morphologic feature of crucial functional relevance for excitation–contraction coupling in ventricular myocytes are substantially reduced or even absent in a major fraction of atrial myocytes (Brette et al., 2005; Frisk et al., 2014), though this is species-dependent (Yue et al., 2017). This again leads to a considerably altered excitation pattern consisting of a slowly propagating centripetal wave in atrial myocytes as compared to the rapid simultaneous and homogenous excitation pattern in ventricular myocytes (Blatter et al., 2003). In addition, there are considerable differences in the expression of functional key proteins participating in excitation–contraction coupling (Boknik et al., 1999; Lüss et al., 1999, 2000; Walden et al., 2009; Nerbonne, 2014). Consequently, these findings suggest significant structural and functional differences and absence of a perfect analogy of both cell types. Moreover, these atrioventricular differences depend on the investigated species. For instance, the t-tubule system in atrial myocytes is reportedly poorly developed in rats, cats, rabbits, and guinea pigs, but well developed in mice or larger mammals like humans (Yue et al., 2017). Likewise, atrioventricular differences in the expression of critical Ca^2+^ handling proteins are species-dependent (Lüss et al., 1999).

The diverse ultrastructure and ion channel expression are reflected in the differing action potential kinetics between atrial and ventricular myocytes in mammals. In general, the resting membrane potential of atrial myocytes is more depolarized as compared to ventricular myocytes, which may be a result of a decreased I_K1_ function in atrial myocytes (Cordeiro et al., 2015). Both, the upstroke velocity and the amplitude of the atrial action potential are decreased in comparison to ventricular myocytes (Grandi et al., 2011), which is promoted by the depolarized resting potential in atrial myocytes that leads to a slower and more incomplete recovery from inactivation of the cardiac sodium channels. Early repolarization in phase 1 is enhanced in atrial myocytes due to the synergistic activation of the transient K^+^ currents I_to(f)_ and atrial-specific I_Kur_ in atrial myocytes (Nerbonne and Kass, 2005). The voltage-level of the action potential plateau in phase 2 is more hyperpolarized in atrial myocytes due to a higher cumulative driving force of repolarizing K_v_ populations. Differences in the action potential duration (APD) seem to be species-dependent, but in higher mammals, the atrial action potential is abbreviated (Burashnikov et al., 2014).

When compared to human action potentials, typical murine ventricular action potentials are significantly abbreviated and exhibit a short plateau phase that starts at more negative voltage levels (Ahrens-Nicklas and Christini, 2009). A large I_to_ in murine myocytes outweighs the depolarizing I_Ca_ and therefore reduces the plateau voltage level, whereas the human I_to_ is less dominant. Taken together, electrophysiological parameters differ between atrial and ventricular myocytes and between different species, as reflected in the characteristic referring action potential shapes.

To account for the significant atrioventricular differences, some recent studies applied computational approaches to mimic atrial electrophysiology to investigate mechanisms of atrial arrhythmia (Zhang et al., 2005; Maleckar et al., 2009; Grandi et al., 2011; Ni et al., 2017). However, despite great advancement during the past decade, computational models are based on assumptions and thus still differ from the genuine atrial electrophysiology, which might particularly apply to complex and stochastic parameters like proarrhythmic afterdepolarizations or atrial fibrillation, that result from a multitude of underlying processes and interactions.

Since murine models are frequently used in translational research on atrial arrhythmia, but data on the manifestation of proarrhythmic afterdepolarizations in atrial myocytes and their differences to ventricular myocytes is sparse, we investigated afterdepolarizations and their molecular bases in atrial and ventricular myocytes in a murine model. The obtained findings might carry several implications for contemporary and future studies investigating atrial arrhythmia in murine models.

## Materials and Methods

### Genetic Background of the Murine Model

Investigated wild-type mice were on a combined genetic background (CD-1 and C57BL/6) as previously described (Bögeholz et al., 2015, 2016) at the age of 7.5–12.5 weeks. The applied experimental methods confirmed to the instructions of Directive 2010/63/EU of the European Parliament on the protection of animals used for scientific purposes and were approved by the local authorities (Landesamt für Natur, Umwelt und Verbraucherschutz NRW; permission number: 84-02.05.50.17.002).

### Isolation of Atrial and Ventricular Cardiomyocytes

The methods to isolate atrial and ventricular cardiomyocytes have been reported in former studies (Bögeholz et al., 2016, 2017). In brief, mice were euthanized by carbon dioxide inhalation and excised hearts were retrogradely perfused via the aorta using a modified Langendorff apparatus. For isolation of ventricular myocytes, hearts were perfused for 6 min ± 15 s on 37°C with a perfusion solution containing (in mM): NaCl 113, KCl 4.7, KH_2_PO_4_ 0.6, Na_2_HPO_4_ 0.6, MgSO_4_ 1.2, NaHCO_3_ 12, KHCO_3_ 10, taurine 30, HEPES 10, glucose 11.1, BDM 10, heparin 14.3 IE/ml, collagenase type II 190 units/ml (Worthington Biochemical Corporation), protease from streptomyces griseus type XIV 0.5 units/ml (Sigma Aldrich), pH = 7.4. Ventricular tissue was dissected below the atrioventricular valves to avoid contamination of the ventricular cell suspension by atrial myocytes. Enzymatic degradation was inhibited by application of new born calf serum and Ca^2+^ was supplemented gradually to 1 mM. To isolate atrial cardiomyocytes, hearts were perfused for 3 min with a perfusion solution containing (in mM): NaCl 136, KCl 5.4, CaCl_2_ 0.2, MgCl_2_ 1, Na_2_HPO_4_ 0.33, HEPES 5, and glucose 11.1, pH = 7.4. Then, a Ca^2+^ depleted perfusion solution was applied for 3 min, followed by enzymatic degradation of the conjunctive tissue for 21 ± 5 min by a Ca^2+^ containing solution with collagenase type II (90 U/mL), bovine serum albumin (1 mg/ml), CaCl_2_ (26.7 nM) and taurine (20 mM). Atria were cut above the atrioventricular valves prior to tissue disruption. Similar to ventricular myocytes, enzymatic degradation was inhibited by new born calf serum and Ca^2+^ was supplemented to a final concentration of 12.5 μM. Atrial myocytes were kept in the following cooled storage solution (in mM): KCl 25, KH_2_PO_4_ 10, L-aspartic acid potassium salt 10, L-glutamic acid potassium salt 100, MgSO_4_ 2, taurine 20, EGTA 0.5, creatine 5, HEPES 5, glucose 20, bovine serum albumin 1 mg/ml, pH = 7.2.

### Two-Dimensional Planimetric Cell Area

Quantification of the two-dimensional planimetric cell area was performed in suspensions of non-fixed atrial and ventricular myocytes using a Photometrics CoolSNAP HQ2 camera on a Nikon eclipse TI-E microscopy system. Quantification was performed with the NIS-Elements AR v. 4.13.05 (Nikon) imaging software.

### Cellular Electrophysiology

For whole-cell patch-clamp experiments, cells were transferred to the stage of an inverted microscope (IX 50, Olympus) and investigated using an EPC 800 amplifier connected to an InstruTECH ITC-18 interface and a MS computer with the PatchMaster v2x53 software (all HEKA, Bellmore, NY, United States) (Bögeholz et al., 2015, 2016). The tip resistance of patch pipettes (GB150TF-8P, Science Products, Hofheim, Germany) was 4 ± 1 MΩ.

Current-clamp measurements were conducted in the perforated patch configuration using amphotericin B (281 μM). The basal extracellular solution contained (in mM): NaCl 136, NaH_2_PO_4_ 0.33, KCl 5.4, CaCl_2_ 1.0, MgCl_2_ 1.0, HEPES 10, Glucose 11.1, pH = 7.4. During the protocol, isoproterenol (1 μM) was applied via a rapid extracellular perfusion system. The intracellular solution contained (in mM): NaCl 5.0, KCl 90.0, KOH 35.0, EGTA 1.0, MgATP 2.5, HEPES 5.0, pH = 7.4.

For voltage-clamp measurements of the voltage-gated L-type Ca^2+^ current (I_Ca_), the ruptured patch configuration was used. Activation of the voltage-gated Na^+^ current was prevented by using a holding potential of -40 mV. I_Ca_ was elicited via depolarizing square wave pulses ranging from -30 to +40 mV each lasting for 400 ms. The following solutions were used (in mM): *extracellular*: NaCl 136.0, KCl 5.4, HEPES 10.0, MgCl_2_ 1.0, NaH_2_PO_4_ 0.33, CaCl_2_ 1.0, Glucose 11.1, pH = 7.4. *Intracellular*: CsCl 120.0, TEA-Cl 10.0, NaCl 10.0, HEPES 20.0, MgATP 5.0, cAMP 0.05, pH = 7.2.

To measure the Na^+^/Ca^2+^-exchanger inward current (I_NCX_), cells were held at -80 mV and depolarized three times to +10 mV. After 10 s of rest, the cells were rapidly perfused with caffeine (10 mM). Extracellular solution contained (in mM): NaCl 140, KCl 4, HEPES 10, MgCl_2_ 1, BaCl_2_ 0.1, 4-amino pyridine 5, glucose 11.1, CaCl_2_ 1, pH = 7.4. Intracellular solution contained (in mM): EGTA 0.02, GTP-Tris 0.1, K-aspartate 92, KCl 48, HEPES 10, MgATP 1, Na_2_ATP 4, pH = 7.2. Measurements were not corrected for liquid junction potentials (-3.3 mV, calculated with Clampex 9.2.1.9, Molecular Devices, San Jose, CA, United States).

### Statistical Analysis

Numerical data are given as means ± standard error of the mean (SEM), or in case of non-normal distributed data, as the median value with the interquartile range (IQR) between the 25th and 75th percentile (Q3–Q1). In box plot figures, the boxes represent the data between 25th and 75th percentile, whiskers data between the 10th and 90th percentile, horizontal lines give the median value, whereas the mean value is shown by the small square. For direct comparison between atrial and ventricular myocytes, the unpaired two-tailed Student’s *t*-test or the Mann–Whitney Rank Sum test was performed, where appropriate. Repeated measurements were analyzed using a two-way repeated measures ANOVA test followed by a Holm–Sidak *post hoc* test. Proportions were compared with a Fisher’s exact test. *P*-values < 0.05 were considered statistical significant.

## Results

### Reduced Cell Area and Cell Capacitance in Murine Atrial Versus Ventricular Myocytes

**FIGURE 1:**
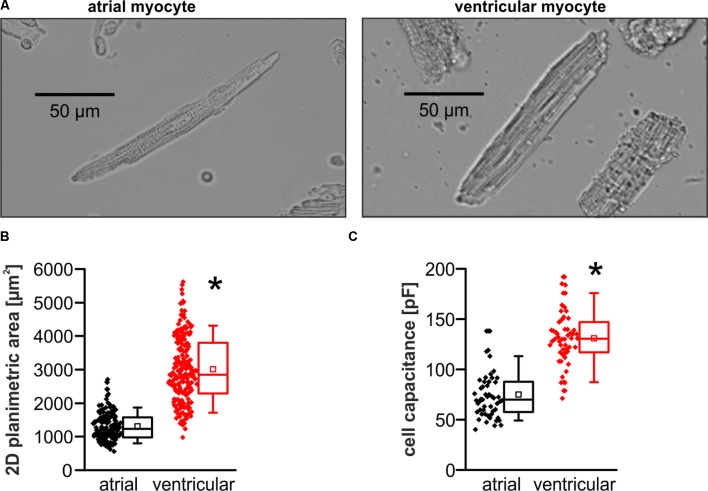
Basic differences in morphometry between atrial and ventricular myocytes. **(A)** Exemplary microscopic images demonstrate morphological differences between murine atrial and ventricular myocytes. Atrial myocytes exhibit a smaller 2D cell area compared to ventricular myocytes **(B)** and consistently a reduced electrical cell capacitance **(C)**. ^∗^*p* < 0.05 atrial vs. ventricular.

Murine atrial myocytes exhibited a fusiform shape and reduced cross-striation in contrast to the rectangular shaped clearly striated ventricular myocytes in microscopic images (**Figure 1A**). The planimetric cell area was significantly smaller in atrial compared to ventricular myocytes (planimetric cell area in μm^2^; atrial myocytes: 1227 [982–1574]; *n* = 154/4 cells/animals; ventricular myocytes: 2852 [2282–3808]; *n* = 208/4 cells/animals; *p* < 0.05) (**Figure 1B**). Consistently, the electrical cell capacitance as a measure for cell size was smaller in atrial myocytes compared to ventricular myocytes (electrical cell capacitance in pF; atrial myocytes: 73.8 ± 3.3; *n* = 49/18 cells/animals; ventricular myocytes: 131.6 ± 3.9; *n* = 54/19 cells/animals; *p* < 0.05) (**Figure 1C**).

### No Protective Action Potential Shortening in Murine Atrial Myocytes

**FIGURE 2:**
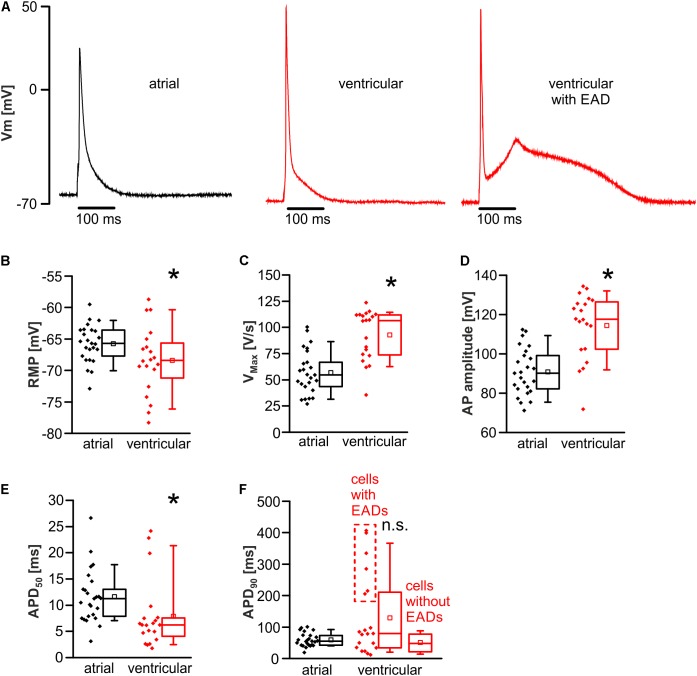
Distinct action potential kinetics in atrial versus ventricular myocytes. **(A)** Representative action potentials in atrial (left) and ventricular cardiomyocytes without (mid) and with (right) an EAD. Atrial myocytes exhibited a depolarized resting membrane potential (RMP) **(B)**, a decreased maximal action potential (AP) upstroke velocity (V_max_) **(C)**, a decreased AP amplitude **(D)**, and prolonged action potential duration to 50% decay (APD_50_) **(E)**. **(F)** Though a fraction of ventricular cells exhibited incessant EADs leading to AP prolongation (raw values within the dashed box), the average APD_90_ was unaltered between both cell types. ^∗^*p* < 0.05 atrial vs. ventricular; n.s., non-significant.

Action potential kinetics were evaluated during 1 Hz pacing after establishing steady state conditions at room temperature (22 ± 2°C) (**Figure 2A**). The resting membrane potential was significantly hyperpolarized in ventricular myocytes when compared to atrial myocytes (resting membrane potential in mV; atrial myocytes: -65.7 [-68.0 to -63.6]; *n* = 25/12 cells/animals; ventricular myocytes: -68.4 [-71.9 to -65.3]; *n* = 20/12 cells/animals; *p* < 0.05) (**Figure 2B**). The maximum upstroke velocity (V_max_) of the action potential as a measure of I_Na_ availability was increased in ventricular vs. atrial myocytes (V_max_ in V/s; atrial myocytes: 57 ± 4.2; *n* = 25/12 cells/animals; ventricular myocytes: 92.8 ± 5.4; *n* = 20/12 cells/animals; *p* < 0.05) (**Figure 2C**). Also, the amplitude of the action potential was increased in ventricular myocytes (action potential amplitude in mV; atrial myocytes: 90.9 ± 2.4; *n* = 25/12 cells/animals; ventricular myocytes: 114.4 ± 3.7; *n* = 20/12 cells/animals; *p* < 0.05) (**Figure 2D**). The APD was quantified as time to 50 and 90% decay (APD_50_ and APD_90_). APD_50_ was significantly prolonged in atrial myocytes (APD_50_ in ms; atrial myocytes: 11.3 [7.7–13.8]; *n* = 25/12 cells/animals; ventricular myocytes: 6.2 [3.8–7.6]; *n* = 20/12 cells/animals; *p* < 0.05) (**Figure 2E**). Six of 20 ventricular myocytes exhibited ongoing EADs mediating a substantial and simultaneously unstable prolongation of the APD_90_ measured in these myocytes (**Figure 2F**). Even when including those cells with EADs in the analysis, APD_90_ was not statistically different between atrial and ventricular myocytes (APD_90_ in ms; atrial myocytes: 55.3 [42.4–73.9]; *n* = 25/12 cells/animals; ventricular myocytes: 79.2 [34.2–212.8]; *n* = 20/12 cells/animals; *p* > 0.05) (**Figure 2F**). Since EAD-deformed action potentials cannot be considered as normal action potentials, they were then excluded from the analysis, again resulting in no difference in APD_90_ between both cell types (APD_90_ in ms; atrial myocytes: 55.3 [42.4–73.9]; *n* = 25/12 cells/animals; ventricular myocytes: 49.3 [23.6–80.7]; *n* = 14/10 cells/animals; *p* > 0.05). Representative recordings of an atrial and ventricular action potential as well as a ventricular action potential deformed by an EAD are presented in **Figure 2A**.

### Reduced Translation of DADs Into Spontaneous Action Potentials in Atrial Myocytes

**FIGURE 3:**
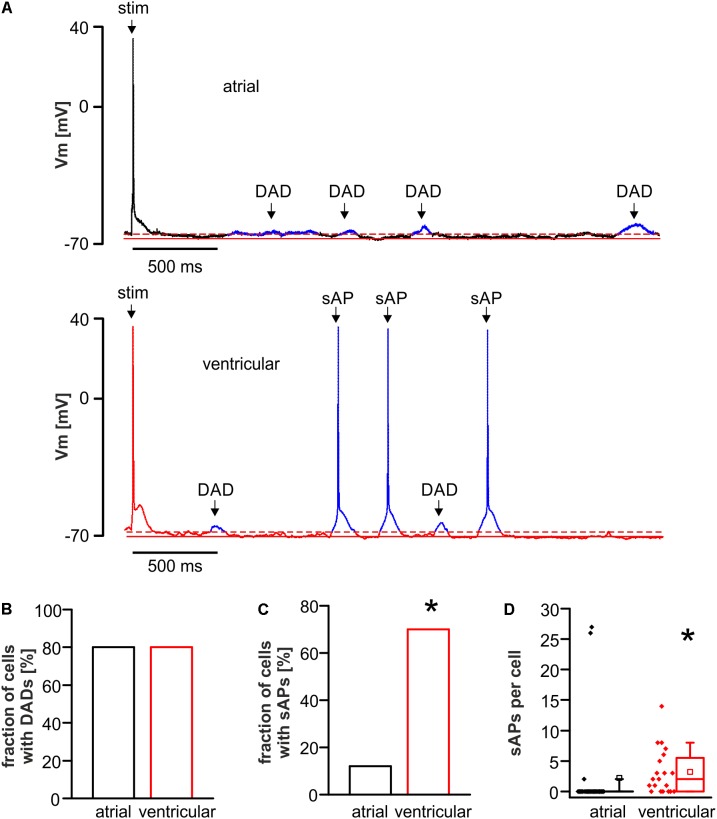
DADs and spontaneous action potentials (sAPs) in atrial and ventricular cardiomyocytes. **(A)** Representative tracings with a stimulated action potential (stim) of atrial (upper panel) and ventricular (lower panel) myocytes exhibiting DADs in the resting period. Ventricular cells did also exhibit translation of DADs into sAPs. While the fraction of cells with DADs was unaltered **(B)**, the fraction of cells with sAPs **(C)** and the average number of sAPs per cell **(D)** was increased in ventricular compared to atrial myocytes. ^∗^*p* < 0.05 atrial vs. ventricular.

The susceptibility toward proarrhythmic afterdepolarizations was evaluated in atrial and ventricular myocytes using the same provocation protocol for both cell types, comprised of abrupt changes between various pacing frequencies and isoproterenol application as established previously with minor modifications (Bögeholz et al., 2015, 2016). In detail, the protocol consisted of 20 stimuli per cycle at 5 Hz; 2 Hz, 1 Hz; 0.5 Hz; 0.25 Hz, and 0.125 Hz followed by 3 min rest. Finally, 1 μM isoproterenol was added during steady-state 1 Hz pacing (1 min.) followed by 20 stimuli at 5 Hz, 2 Hz, 1 Hz, and 0.5 Hz resulting in a total protocol duration of 10 min and 28 s. Delayed afterdepolarizations (DADs) were defined as low-amplitude depolarizations exceeding the diastolic membrane potential for more than 2 mV (**Figure 3A**). Spontaneous action potentials (sAPs) defined as fully developed action potentials arising from a DAD and exceeding -10 mV threshold, were counted (**Figure 3A**).

The fraction of cells exhibiting DADs was similar in both cell types (fraction of cells with DADs in %; atrial myocytes: 80.0; *n* = 25/12 cells/animals; ventricular myocytes: 80.0; *n* = 20/12 cells/animals; *p* > 0.05) (**Figure 3B**). sAPs occurred in both cell types, but the fraction of cells with sAPs was significantly higher in ventricular myocytes (fraction of cells with spontaneous AP in %; atrial myocytes: 12.0; *n* = 25/12 cells/animals; ventricular myocytes: 70.0; *n* = 20/12 cells/animals; *p* < 0.05) (**Figure 3C**). The median number of sAPs per cell was significantly decreased in atrial versus ventricular myocytes (**Figure 3D**).

### Absence of Early Afterdepolarizations in Atrial Myocytes

**FIGURE 4:**
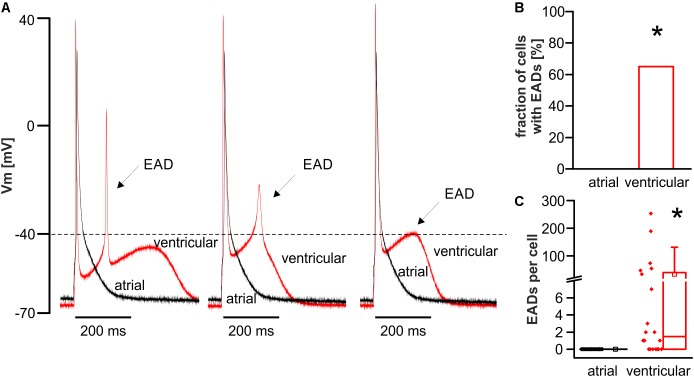
Occurrence of EADs in ventricular but not atrial cardiomyocytes. **(A)** Representative tracings show various shapes of EADs in ventricular myocytes (red tracing) when the membrane potential of the plateau first falls short of and then exceeds –40 mV threshold. The EAD waveforms ranged between a spike-like shape (left) and a prolonged slew rate (right). No EADs occurred in any atrial myocyte (black tracing). The majority of ventricular cells **(B)** exhibited multiple EADs **(C)**. ^∗^*p* < 0.05 atrial vs. ventricular.

Throughout the above stated protocol, the occurrence of early afterdepolarizations (EADs) was quantified in atrial and ventricular myocytes. EADs were defined as re-upstrokes of the membrane potential during repolarization of a stimulated action potential, if the membrane potential of the action potential plateau shortly falls of and then exceeds -40 mV threshold (**Figure 4A**). EADs showed various shapes as illustrated in **Figure 4A**.

Under the experimental conditions chosen, no EAD occurred in any atrial myocyte, whereas EADs occurred frequently in ventricular myocytes. In detail, the majority of ventricular myocytes exhibited EADs (fraction of cells with EADs in %; atrial myocytes: 0; *n* = 25/12 cells/animals; ventricular myocytes: 65.0; *n* = 20/12 cells/animals; *p* < 0.05; median number of EADs per cell; atrial myocytes: 0.0; *n* = 25/12 cells/animals; ventricular myocytes: 1.5 [0.0–42.8]; *n* = 20/12 cells/animals; *p* < 0.05) (**Figures 4B,C**). The median voltage take-off potential for EADs was -44.8 mV [-51.6 to -42.2].

### Reduced L-Type Ca^2+^ Current in Murine Atrial Myocytes

**FIGURE 5:**
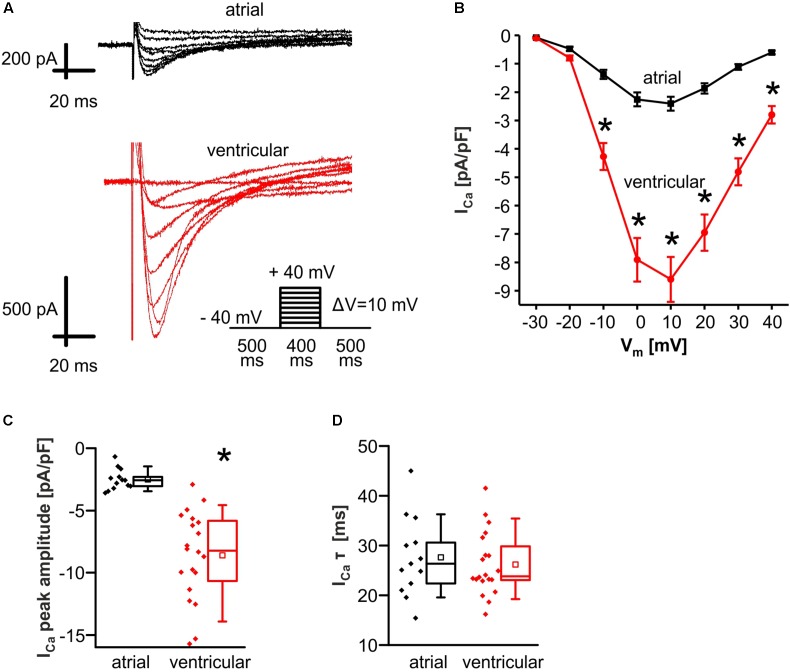
Reduced L-type Ca^2+^ current (I_Ca_) in atrial versus ventricular myocytes. **(A)** Original tracings of I_Ca_ in atrial (upper panel) and ventricular (lower panel) myocytes. The applied stimulation protocol is depicted by the inset scheme. I_Ca_ was significantly reduced in atrial compared to ventricular myocytes over a voltage range between –10 mV and +40 mV as shown in the I–V-curve representing the mean values **(B)**. **(C)** Median values of I_Ca_ peak amplitude in response to a voltage step to +10mV. **(D)** I_Ca_ inactivation kinetics were unaltered between both cell types. ^∗^*p* < 0.05 atrial vs. ventricular [two-way repeated measures ANOVA for **(B)**].

To further investigate the molecular mechanisms of EAD and DAD, the depolarizing L-type Ca^2+^ current (I_Ca_) was measured in atrial and ventricular myocytes (**Figure 5A**). To account for differences in cell size, peak I_Ca_ was normalized to the cell capacitance as a measure of cell surface area.

I_Ca_ peak amplitude was significantly reduced in atrial myocytes as compared to ventricular myocytes (peak amplitude of I_Ca_ in response to a square wave pulse to +10 mV in pA/pF; atrial myocytes: 2.6 [2.0–3.1]; *n* = 13/3 cells/animals; ventricular myocytes: 8.2 [5.7–11.0]; *n* = 20/4 cells/animals; *p* < 0.05) (**Figures 5B,C**). Inactivation of I_Ca_ was estimated by fitting a single exponential decay function to I_Ca_ tracings in response to a voltage step to +10 mV. I_Ca_ inactivation was unaltered between both cell types (τ in ms; atrial myocytes: 27.7 ± 2.2; *n* = 13/3 cells/animals; ventricular myocytes: 26.2 ± 1.4; *n* = 20/4 cells/animals; *p* > 0.05) (**Figure 5D**).

### Smaller NCX Inward Current but Increased SR Ca^2+^ Load in Atrial Versus Ventricular Myocytes

The depolarizing NCX inward current (I_NCX_; extrusion of 1 Ca^2+^ from the cell in exchange for 3 Na^+^) is thought to promote the occurrence of EAD and DAD. Thus, I_NCX_ was investigated in atrial and ventricular myocytes.

**FIGURE 6:**
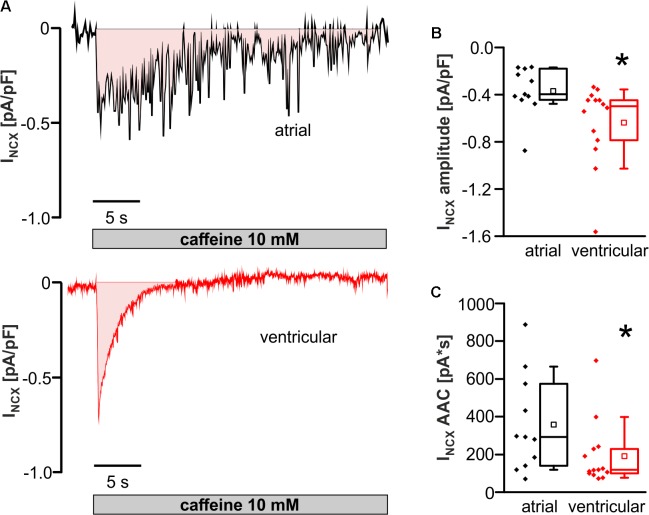
Caffeine-induced NCX inward current (I_NCX_) in atrial versus ventricular myocytes. **(A)** Original tracings of caffeine-induced NCX inward current (I_NCX_) in atrial (upper panel) and ventricular (lower panel) cells. **(B)** I_NCX_ amplitude in response to caffeine was significantly reduced in atrial versus ventricular myocytes. **(C)** Integrated I_NCX_ from the area above the current curve (pink colored area). ^∗^*p* < 0.05 atrial vs. ventricular.

I_NCX_ peak amplitude was significantly decreased in atrial compared to ventricular myocytes (I_NCX_ amplitude in pA/pF; atrial myocytes: -0.40 [-0.44 to -0.18]; *n* = 11/3 cells/animals; ventricular myocytes: -0.50 [-0.81 to -0.44]; *n* = 14/3 cells/animals; *p* < 0.05) (**Figures 6A,B**). The area above the curve (AAC) of the inward current up to baseline (**Figure 6A**) is considered as a measure for the sarcoplasmic Ca^2+^ content. AAC was larger in atrial than ventricular myocytes (AAC of I_NCX_ in pA^∗^s; atrial myocytes: 293 [139–574]; *n* = 11/3 cells/animals; ventricular myocytes: 118.0 [98–232]; *n* = 14/3 cells/animals; *p* < 0.05) (**Figure 6C**).

## Discussion

The use of murine models is widespread in experimental research, due to facilitated accessibility to genetic manipulation, short reproduction cycles and comparable low housing and breeding costs. Hence, many experimental findings that describe molecular mechanisms of arrhythmia, originate from single cell experiments in murine models. However, the translational value of findings derived from murine models is often called into question (Kaese and Verheule, 2012; Bögeholz et al., 2014; Nerbonne, 2014), since major electrophysiological properties differ considerably between humans and mice. Furthermore, the majority of proarrhythmic mechanisms were derived from ventricular murine cardiomyocytes, despite the higher prevalence of atrial arrhythmia and the enormous clinical attention toward supraventricular arrhythmia, such as atrial fibrillation. This might be due to the robustness and ease of isolation of ventricular myocytes facilitating experimental approaches to investigate the cellular electrophysiology. However, due to ultrastructural and functional differences between ventricular and atrial cardiomyocytes the proneness toward proarrhythmic afterdepolarizations might differ between both cell types. Consequently, in face of these atrioventricular differences, several studies investigated murine models, particularly on the atrial level, using isolated cardiomyocytes (Lemoine et al., 2011; Faggioni et al., 2014), multicellular preparations (Bao et al., 2016; Syeda et al., 2016) and whole-heart approaches (Wan et al., 2016; Chang et al., 2017; Wang et al., 2017) to evaluate translational concepts in the generation of atrial fibrillation. We here present direct evidence that the manifestation of proarrhythmia in the form of the occurrence of EAD and DAD considerably differs between atrial and ventricular murine myocytes, which carries several potential implications for arrhythmia research in murine models, especially on the atrial level.

### Protection Against EADs in Atrial Myocytes

In line with previous studies on rats (Walden et al., 2009), murine atrial myocytes were significantly smaller and exhibited a fusiform shape with reduced cross-striation as compared to the larger and rectangular ventricular myocytes. The applied provocation protocol in our study was sufficient to elicit frequent DADs and several sAPs in both cell types. However, though the majority of ventricular myocytes exhibited frequent EADs, no single EAD occurred in any investigated atrial myocyte. Consistently, other studies found that genetic or pharmacological interventions that prolong the APD are required to elicit EADs on atrial level in murine models (Li et al., 2009; Qin et al., 2012). However, these findings were not compared to ventricular myocytes. The observed protection against EADs in this study is not a result of an abbreviated action potential, because APD_90_ only tended to be increased in ventricular myocytes due to a fraction of cells that exhibited EADs (**Figure 2F**), but overall, the APD_90_ was not statistically different between both cell types. APD_50_ was prolonged in atrial myocytes, but this likely does not promote EADs, since EADs typically occur at a voltage below the level reached at APD_50_. The findings with regard to the APDs are in good accordance with previous findings from our group when comparing data from FVB/N wild-type controls, published recently in two separate studies, demonstrating APD_50_ values of 8.75 ± 0.8 ms and 4.6 ± 0.5 ms and APD_90_ values of 38.7 ± 2.7 ms and 32.5 ± 4.4 ms for atrial (Seidl et al., 2017) and ventricular (Schulte et al., 2016) myocytes, respectively. Of note, these findings on the action potential kinetics of atrial and ventricular myocytes do not apply to higher mammals or even human action potential kinetics, since human atrial cardiomyocytes exhibit a substantially prolonged action potential plateau (APD_90_ ∼ 300 ms) (Christ et al., 2016). Likewise, the human ventricular action potential is considerably prolonged as compared to mouse models (APD_90_ ∼ 450–500 ms) (Johnson et al., 2018). These action potential kinetics may allow EADs in human atrial myocytes rather than in murine atrial myocytes. Nonetheless, vigorous provocation conditions like strong inhibition of repolarizing K^+^ channels, resulting in a significant APD prolongation, is required to elicit EADs in human atrial myocytes (Olson et al., 2006). If the APD is prolonged by vigorous pharmacological interventions, EADs can even be elicited in murine atrial trabeculae (Zhou and Liu, 1995). Hence, the protection against EADs in murine atrial myocytes can be overridden by aggressive pharmacological interventions. However, murine atrial myocytes are still less susceptible to EADs as compared to ventricular myocytes, which is of relevance for studies on atrial arrhythmia using murine models.

What is the underlying mechanism of protection against EADs in murine atrial myocytes? Since the classical mechanism in the generation of an EAD consists of a reactivation of I_Ca_ or enhanced NCX-mediated inward current (Volders et al., 1997), the prominent reduction of both, as observed in this study, suggests a plausible explanation for the protection against EADs in atrial myocytes. Beyond that, I_Ca_ and I_NCX_ may be functionally coupled. Our group has shown that the reduced NCX function in murine ventricular myocytes of homo- and heterozygous NCX knockout mouse models is accompanied by a reduction of I_Ca_ (Pott et al., 2007; Bögeholz et al., 2015). In ventricular myocytes, the underlying mechanism seems to consist of an increased subsarcolemmal Ca^2+^ concentration due to reduced NCX-mediated Ca^2+^ extrusion capacitance, which enhances Ca^2+^-dependent inactivation of I_Ca_. Thus, the inhibitory interaction between I_NCX_ and I_Ca_ may synergistically protect against the occurrence of EADs. The same mechanism could apply to atrial myocytes, although this needs to be further investigated, since the functional coupling of I_NCX_ and I_Ca_ may be different in atrial myocytes due to a diverse ultrastructure. However, there may be also differences in other depolarizing and repolarizing ion currents that may reduce the proneness toward EADs in atrial myocytes. Recently, Edwards et al. (2014) proposed a role for non-equilibrium sodium current (I_Na_) reactivation in the generation of EADs in mice, based on the much faster action potential repolarization velocity in comparison to higher mammals. A role for I_Na_ reactivation is also likely in our study since the median take-off potential of EADs in ventricular myocytes was -44.8 mV, which is below the I_Ca_ activation range. Though we did not directly measure I_Na_ kinetics in our study, the reduced maximal action potential upstroke velocity and the reduced action potential amplitude in atrial vs. ventricular myocytes are both indicators for a reduced Na^+^ channel availability. One possible cause is the relatively depolarized resting membrane potential in atrial myocytes, which will reduce the availability of Na^+^ channels and driving force for I_Na_ in atrial versus ventricular myocytes. Furthermore, Edwards et al. (2014) demonstrated that I_Na_ reactivation enhances with increasing velocity of repolarization. Since atrial myocytes had a rather triangular action potential shape along with increased APD_50_, the reduced repolarization velocity in atrial versus ventricular myocytes may thus additionally contribute to the protection against EADs in murine atrial myocytes. Interestingly, in their study Edwards et al. (2014) also demonstrated in a computational model that ablation of I_NCX_ was able to eliminate EADs and that I_Ca_ was also reactivated to a small extent during EADs secondary to I_Na_. Thus, the reduction of both currents in atrial myocytes should contribute to the reduced propensity to EADs in atrial mouse cardiomyocytes as observed in our study.

### DADs and Spontaneous Action Potentials in Atrial Versus Ventricular Myocytes

Both atrial and ventricular myocytes exhibited frequent DADs on average in 4 out of 5 (80%) cells during the applied provocation protocol, which contains abrupt changes in pacing cycle lengths and additional catecholaminergic stimulation. Hence, the conditions were sufficient to elicit proarrhythmia in both cell types. Since DADs result from spontaneous Ca^2+^ release from the sarcoplasmic reticulum (SR), the observed increase of SR Ca^2+^ load as assessed by the integrated NCX inward current in atrial compared to ventricular myocytes may promote the occurrence of DADs in atrial myocytes. An increased SR Ca^2+^ load of atrial compared to ventricular myocytes in smaller rodents has been demonstrated previously (Walden et al., 2009). However, the occurrence of sAPs as the final proarrhythmic consequence of DADs was significantly reduced in atrial compared to ventricular myocytes. This finding might be surprising at first sight, since the resting membrane potential in atrial myocytes is more depolarized as compared to ventricular myocytes, which should facilitate the translation of DADs in sAPs. However, I_NCX_ peak current as the key translator of spontaneous Ca^2+^ release events into sAPs is significantly reduced in atrial myocytes, which could potentially explain for the reduced occurrence of sAPs in atrial myocytes. Next to the reduced I_NCX_ peak current, two potential alternative alterations may protect against the occurrence of afterdepolarizations in atrial myocytes. These could be a reduced availability of I_Na_ and/or increased repolarization reserve. Although these components were not directly measured, the upstroke velocity of the action potential is slowed in atrial versus ventricular myocytes, which can be considered as a proxy measure for reduced I_Na_ as the final translator of I_NCX_ mediated membrane depolarization into a sAP. This may also be a result of the depolarized resting membrane potential in atrial myocytes. In principle, atrial myocytes could also be protected against afterdepolarizations by an increased repolarization reserve potentially mediated via increased I_K1_. However, since I_K1_ is the major hyperpolarizing current in diastole and the resting membrane potential is depolarized in atrial myocytes, the latter possibility seems less likely. Not at least, the substantially diverse ultrastructure of the SR and its altered coupling to the plasma membrane may also contribute to the reduced translation of DADs into sAPs (Frisk et al., 2014).

### Translational Perspective on Murine Models in Research on Atrial Arrhythmia

Early and delayed afterdepolarizations are thought to promote the occurrence of human atrial arrhythmia, such as atrial fibrillation (Patterson et al., 2006; Beavers et al., 2013). Mice are commonly used as models for the investigation of basic proarrhythmic mechanisms and therapeutic strategies for treatment of atrial fibrillation. Thus, it is of relevance to characterize cellular proarrhythmic substrates like EADs and DADs and existing specific differences between atrial and ventricular myocytes in the mouse. Our findings show that the occurrence of EADs, DADs, and sAPs significantly differs between atrial and ventricular myocytes. Thus, proarrhythmia in both cell types cannot be considered equal. Thus, a direct translation of the ventricular proarrhythmia to the atrial level is questionable. Although basic causes for afterdepolarizations (i.e., I_NCX_ and reactivation of I_Ca_ and I_Na_) might be similar, the relative quantitative contribution and their functional interaction seem to differ in an environment of a diverse expression of other ion channel populations and the referring cellular microarchitecture. Together, these differences finally result in the distinct probability for the occurrence of afterdepolarizations in atrial and ventricular myocytes. This finding is of relevance for the evaluation of antiarrhythmic strategies targeting atrial arrhythmia at least when using murine models on the single cell and multicellular level. However, it is debatable, how good the current data situation actually is regarding a direct comparison of atrial and ventricular proarrhythmia in other species – particularly in humans, which is complicated by the availability of material to examine. In view of this limited availability – especially of human myocardial tissue – computational modeling of atrial electrophysiology is a promising alternative approach, but its reliability strongly depends on the completeness of the integrated data derived from experimental research (Grandi et al., 2010; Bers and Grandi, 2011; Pandit et al., 2011). There are computational models of human atrial myocytes that simulate physiological and pathophysiological conditions in terms of chronic atrial fibrillation based on ventricular models (Grandi et al., 2011). Atrial electrophysiology is simulated by adopting several ion current functions in part based on the findings of studies that reported the protein expression but not function (Wang et al., 1996). Furthermore, some parameters regarding functional ion channel properties are derived from single references, since experimental data from human atrial versus ventricular myocytes is sparse.

Occurrence of whole-heart arrhythmias is even more complex than basic cellular mechanisms of arrhythmia, since its initiation depends on the stochastic coincidence of cellular arrhythmic events in relation to the regular heart beat. The occurrence and proarrhythmic significance of afterdepolarizations in isolated cardiomyocytes differs from those of cardiomyocytes that are embedded within the myocardial syncytium. Neighboring cardiomyocytes, coupled via gap junctions, might serve as a current-sink and thus counterbalance proarrhythmic alterations of the membrane potential of a single myocyte, which reduces the probability for a sAP to occur. Thus, depolarization of a critical number of adjacent cardiomyocytes is required to initiate an action potential on the multicellular level (Rubart and Zipes, 2005). Furthermore, the likelihood for initiation of sustained whole-heart arrhythmia does not only depend on the mere number of premature ventricular complexes (PVCs), but also on the timing of the PVC in relation to the prior heartbeat as reflected in the typical short-long-short initiation pattern of torsade-de-pointes tachycardias (Kay et al., 1983) or its short-coupled variant (Leenhardt et al., 1994). Beyond the initiation of whole-heart arrhythmia, its perpetuation is even more complex and underlies a multitude of factors including the anatomical structure, pathophysiological alterations like myocardial scars and the influence of antiarrhythmic drug or catheter-based therapy.

### Limitations

Experiments were conducted at room temperature. Thus, functional parameters may differ, when these experiments are performed at physiological temperatures. The current study was performed on cardiomyocytes isolated from a non-diseased mouse model. It is possible that remodeled atrial cardiomyocytes from certain disease-models are not protected against the occurrence of EADs and DADs. However, especially murine models of atrial fibrillation are very diverse in showing shortened, unchanged or even prolonged APDs as a reflection of a heterogeneous underlying electrophysiology (Riley et al., 2012) and thus may not always reflect the pathophysiology of human atrial fibrillation where action potentials are usually prolonged. Consequently, every model should be evaluated individually regarding its validity and limitations.

## Conclusion

Taken together, we have demonstrated that the manifestation of proarrhythmic afterdepolarizations like EADs and the development of sAPs from DADs differs between atrial and ventricular myocytes in mice. This has to be considered when using murine models for translational research particularly with regard to atrial arrhythmia like atrial fibrillation. When using other animal, *in vitro* or computational models, we should critically scrutinize the underlying electrophysiological features and other fundamental aspects of the cell types before transferring findings derived from the ventricular level to atrial level, especially with regard to arrhythmia.

## Author Contributions

NB and JS designed the study. NB and PP acquired the data. NB, PP, DD, and JS performed the statistical analysis. NB, PP, and JS wrote the manuscript. DD, GF, JG, CP, FM, and LE critically revised the manuscript. All authors contributed substantially to the interpretation of the data and approved the final version of the manuscript.

## Conflict of Interest Statement

The authors declare that the research was conducted in the absence of any commercial or financial relationships that could be construed as a potential conflict of interest. The reviewer DF and handling Editor declared their shared affiliation.
